# On the Role of the Difference in Surface Tensions Involved in the Allosteric Regulation of NHE-1 Induced by Low to Mild Osmotic Pressure, Membrane Tension and Lipid Asymmetry

**DOI:** 10.1007/s12013-012-9340-7

**Published:** 2012-02-14

**Authors:** Vincent Pang, Laurent Counillon, Dominique Lagadic-Gossmann, Mallorie Poet, Jérôme Lacroix, Odile Sergent, Raheela Khan, Cyril Rauch

**Affiliations:** 1 School of Veterinary Medicine and Science, University of Nottingham, College Road, Sutton Bonington, LE12 5RD UK; 2 School of Graduate Entry Medicine and Health, Faculty of Medicine & Health Sciences, University of Nottingham, Derby, E22 3DT UK; 3 EA 4427 SeRAIC, Université Rennes I, IFR 140, 2 Avenue du Professeur Leon-Bernard, 35043 Rennes Cedex, France; 4 Université de Nice-Sophia Antipolis, Transport Ionique Aspects Normaux et Pathologiques, CNRS UMR6097 Faculté des Sciences, Parc Valrose, 06108 Nice Cedex 2, France

**Keywords:** Allosteric switch, Ion channels, Membrane, Surface tension, Endocytosis, Lipid asymmetry

## Abstract

The sodium-proton exchanger 1 (NHE-1) is a membrane transporter that exchanges Na^+^ for H^+^ ion across the membrane of eukaryotic cells. It is cooperatively activated by intracellular protons, and this allosteric regulation is modulated by the biophysical properties of the plasma membrane and related lipid environment. Consequently, NHE-1 is a mechanosensitive transporter that responds to osmotic pressure, and changes in membrane composition. The purpose of this study was to develop the relationship between membrane surface tension, and the allosteric balance of a mechanosensitive transporter such as NHE-1. In eukaryotes, the asymmetric composition of membrane leaflets results in a difference in surface tensions that is involved in the creation of a reservoir of intracellular vesicles and membrane buds contributing to buffer mechanical constraints. Therefore, we took this phenomenon into account in this study and developed a set of relations between the mean surface tension, membrane asymmetry, fluid phase endocytosis and the allosteric equilibrium constant of the transporter. We then used the experimental data published on the effects of osmotic pressure and membrane modification on the NHE-1 allosteric constant to fit these equations. We show here that NHE-1 mechanosensitivity is more based on its high sensitivity towards the asymmetry between the bilayer leaflets compared to mean global membrane tension. This compliance to membrane asymmetry is physiologically relevant as with their slower transport rates than ion channels, transporters cannot respond as high pressure-high conductance fast-gating emergency valves.

## Introduction

**Fig. 1:**
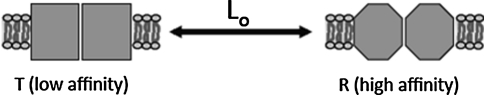
Schematic depiction of the MWC allosteric regulation of NHE-1. The dimeric transporter oscillates between a low and a high affinity form for intracellular protons. In physiological conditions, intracellular acidification will result in the protonation of the high affinity form and trigger the cooperative activation of the system by protons. The allosteric equilibrium constant $L_{0}$ is modulated by the modifications of the transporter by signalling pathways and by changes in the surrounding membrane

The detection and reaction to mechanical forces is central in all biological systems. In the past few years and until very recently [1], the basic knowledge on mechanosensitive proteins has greatly expanded by the cloning and characterization of the underlying molecular entities that appeared to be mainly ion channels [2–7]. As well, an important body of works has described the biophysics of the lipid bilayer surrounding these membrane proteins and related surface pressure in their regulation. Recently, a growing body of evidences has shown that several ion transporters are also sensitive to modifications of membrane tension and composition [8–14]. In this context, the ubiquitous Na^+^/H^+^ exchanger NHE-1, that has been firstly described as a major pH regulator, is also a cell volume regulator as it becomes activated when cells are under hypertonic conditions, resulting in the shuttling of osmotically active sodium ions into the cytoplasm [15]. In addition to this osmosensitivity, NHE-1 can respond directly to mechanical forces applied to the membrane or to changes in lipid packing and cholesterol concentration [12, 13, 16]. The activation of NHE-1 under different conditions was shown to follow a Monod–Wyman–Changeux (MWC) mechanism [12]. In this model NHE-1, as a dimeric protein, oscillates between a low and a high affinity form for protons, making it able to sense acidification in a cooperative manner. This allosteric shift between two conformational states depicted by the $L_{0}$ allosteric equilibrium constant, is reminiscent to the oscillation of a channel between closed and open states (see Fig. 1). In accordance, we have recently shown that $L_{0}$ is modified by osmotic pressure, independently of the signalling pathways known to activate NHE-1, and with an exponential distribution that is similar to that of the open probability of mechanosensitive channels [12]. As well, we showed that crenators or cup-formers, [17] could modify NHE-1 allosteric balance, [12, 18, 19], whilst elimination of PIP2/ERM binding sites did not abolish its mechanosensitivity. Taken together, these results strongly suggested that NHE-1 mechanosensitivity resides in its ability to sense directly membrane differential packing. About 10 years ago the relationship between the cell membrane mechanical properties and endocytosis (i.e., membrane budding) were joined together allowing an understanding of how membrane lipid asymmetry triggers fluid phase endocytosis [20, 21]. Approximately at the same period was suggested that the physical properties of membrane could be a strong modulator of membrane proteins activity [22]. Therefore, although the mean surface tension was the first parameter introduced to describe the theory behind the switch of mechosensitive membrane proteins [23]; we reasoned that the differential compression of lipid leaflets should be taken into account in the present studies. Despite all these similarities with ion channels, secondary ion transporters have much slower rates of ion translocation across biological membranes. Therefore, we can hypothesize that, unlike channels, transporters will be unlikely to work efficiently as emergency high-conductance pressure valves but instead should exhibit smoother responses over longer kinetics [18, 19, 23–25]. In this context, NHE-1 appears as an excellent paradigm to model the modulation of the allosteric constant of a transporter by membrane biophysical constraints. Our model could be then challenged for its physical and biological relevance using our experimental data. Taken together our results show a good adequacy between data obtained from kinetic analyses and the physical description of NHE-1 membrane interactions, provided that it includes membrane asymmetry.

## Results

### Why is it Necessary to Take the Membrane Asymmetry Into Account?

The allosteric switch of NHE-1 in cells is activated when the osmolarity of the extracellular milieu is changed [12]. At first sight, if the cell membrane was considered perfectly symmetrical and thus cells as perfect osmometers, due to the changes in surface tension Laplace’s law should apply. In these conditions basic thermo-chemistry infers the presence of an energy barrier between NHE-1 allosteric states. NHE-1 switching between two states is thus expected to be described by Boltzmann’s relation (i.e., Arrhenius’ Law) under the form: $\sim \exp ( - E/k_{\text{B}} T)$; where *E* characterizes the interaction energy between the osmotic pressure applied, membrane surface tension changes and NHE-1.

If the osmotic pressure is thought to exert its effect on mechanosensitive membrane proteins (as NHE-1) via alteration of lateral mechanical stretch, then the interaction energy can be written as: $E\sim A_{\text{NHE1}} \times \sigma$; where, $A_{\text{NHE1}}$, is the cross-sectional area of NHE-1 and, σ, the surface tension ahead of osmotic changes (we shall assume that the surface tension is low in resting conditions). Applying Laplace’s Law (i.e., assuming cells as perfect osmometer and a spherical cell), the interaction energy can be rewritten as: $E\sim A_{\text{NHE1}} R_{\text{cell}} \Updelta P/2$, where ∆*P* is the pressure difference between the outside and the cytosol and $R_{\text{cell}}$ the cell radius. In this context, by noting $P_{0}$ the resting isotonic pressure, it is expected that the allosteric switch of NHE-1 follows: $\sim \exp [( - A_{\text{NHE1}} R_{\text{cell}} P_{0} /2k_{\text{B}} T) \times (\Updelta P/P_{0} )]$.

For a small percentage change in, $\Updelta P/P_{0}$, the system will only change appreciably if the pre-factor in the exponential function that sets the sensitivity of NHE-1 to osmotic changes (i.e., $A_{\text{NHE1}} R_{\text{cell}} P_{0} /2k_{\text{B}} T$) is sufficiently large. This pre-factor can be estimated. Let us assume that NHE-1 is a dimeric molecule represented as the union of two cylinder-like monomers (Fig. 1) of individual cross-sectional area, $A_{\text{NHE1}} /2$. Providing the molecular weight (MW) of the embedded part of NHE-1 in the membrane: ${\text{MW}}_{\text{NHE1}} \sim 55\,{\text{kDa}}$, and assuming that the MW of the protein is proportional to its volume in first approximation [26] one finds: ${\text{MW}}_{\text{NHE1}} \sim 2 \times (hA_{\text{NHE1}} /2)$. The later relation is true only if all the spatial dimensions are expressed in angstrom units. With $h\sim 5nm$ the cross sectional area of NHE-1 can then be estimated: $A_{\text{NHE1}} \sim {\text{MW}}_{\text{NHE1}} /h\sim 11\,{\text{nm}}^{2}$.

Considering $P_{0} = 280{\text{mOsm}} = 7.1 \times 10^{5} \,{\text{Pa}}$ and a typical cell radius of $R_{\text{cell}} \sim 10 - 20\mu {\text{m}}$, one finds: $A_{\text{NHE1}} R_{\text{cell}} P_{0} /2k_{\text{B}} T\sim 1 - 2 \times 10^{4}$ (at 37°C). This last result differs by about one order of magnitude from experimental data obtained by Lacroix et al. [12]. Indeed this study determined experimentally in living cells that $A_{\text{NHE1}} R_{\text{cell}} P_{0} /2k_{\text{B}} T\sim 2.8 \times 10^{3}$.

**Fig. 2:**
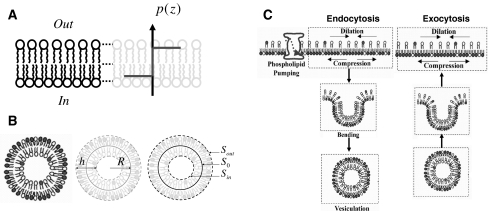
Schematic representation of surface pressure, lipid asymmetry, membrane budding and endocytosis in membranes. **a** Sketch representing the profile of surface pressure within the membrane. **b** Schematic relation between fluid phase endocytosis and the differential packing of lipids. The vesicle radius is, geometrically speaking, inversely proportional to the lipid number asymmetry between leaflets [21]. **c** The phospholipid number asymmetry maintained by the aminophospholipid translocase leads to membrane bending and vesiculation**.** Note that the membrane recycling that occurs in cells (*right panel*), with a size similar to endocytic vesicles, allows the maintenance of the lipid asymmetry. Given the lipid asymmetry in membranes it is supposed that the surface pressure is constant in either leaflet [21]

This discrepancy between the calculated and experimental value has to be related to the presence of the large reservoir of membrane in eukaryotic cells that permits the buffering of osmotic pressure, and related surface tension changes [27–29]. Indeed, without this mechanism, cell membranes would be excessively fragile and a typical membrane surface area dilation as low as ~3% would tear them apart [30]. Thus, understanding NHE-1 regulation by membrane mechanical forces requires integrating the way cells allow their membrane to buffer osmotic challenge as well. This large reservoir buffer is at least in part created by lipid asymmetry, maintained by one or several lipid flippase [31, 32]. This asymmetry, and associated differential lipid packing between membrane leaflets (Fig. 2), is central for creating membrane buds that result in fluid phase endocytosis and membrane recycling [20, 21]. Recently, a model involving the radius of fluid phase vesicle (and related kinetic of membrane endocytosis) in the control of the cytosolic osmotic pressure has been advanced and successfully compared to experimental data [33]. In short this model demonstrates that the difference in osmotic pressures between the inside and outside of cells impacts on the ability of the membrane to form buds. This physical competition between membrane budding and osmotic pressure changes the radius of fluid phase vesicles that, in turn, allows cells to maintain a constant cytosolic pressure up to a certain osmotic threshold [21, 34]. Thus, up to this threshold, the cell membrane preserves a steady mean surface tension [21, 34]. To summarize, the lipid packing asymmetry that is connected to fluid phase endocytosis has to be taken into account to model NHE-1 allosteric activation mediated by changes in osmotic pressure and/or membrane tension.

### Estimation of the Effect of Membrane Mean Surface Tension and Difference in Surface Tensions on the Allosteric Activation of Ion Channels or Ion Transporters

Note that in what follows, the energy of the mismatch between the membrane thickness and that of the membrane protein will not be dealt with as: (i) many excellent papers or reviews exist on the subject (see for example: [22, 25, 35]) and that; (ii) we shall see retrospectively that comparisons between theory and experimental data is satisfactory without involving this parameter.

To develop the relationship between the mean surface tension, endocytosis and the allosteric balance of membrane proteins, one will follow on Robert S. Cantor’s works [22, 36]. First let us consider that NHE-1 in the MWC mechanism can be in two states, a high-affinity state or a low-affinity state. The ratio between the numbers of low to high-affinity states initially is noted by “$L_{0}$”. Upon change in the membrane, this ratio will be altered and noted “*L*”. The ratio between the allosteric states of the membrane protein when the membrane is under stress can be written as (see appendix 1):

1 $$ L/L_{0} = \exp \left( { - \int\limits_{ - h/2}^{h/2} {\left( {p(z) - p_{0} (z)} \right)\left( {A_{\text{low}} (z) - A_{\text{high}} (z)} \right)} {\text{d}}z/k_{\text{B}} T} \right) $$

In Eq. 1, the subscript “0” refers to the initial state, $p(z)$ is the surface pressure across the membrane that varies along the constant thickness, *h*, of the membrane denoted by the *z* axis, and $A_{\text{low,high}} (z)$ the cross-section area of the membrane protein along the same axis in either state, low or high affinity to protons. For the sake of simplicity, one will assume that the surface tension in each leaflet is constant and follows a symmetrical Heaviside-type (step-wise) distribution driven by the difference in surface tensions (i.e., lipid asymmetry) (see Fig. 2a). All together these hypotheses allows one to write: $p(z) - p_{0} (z)\sim (\sigma - \sigma_{0} )/h = {\text{cte}}$. In this case Eq. 1 can be rewritten as:

2 $$ \begin{gathered} L/L_{0} \sim \hfill \\ \exp \left( { - \int\limits_{0}^{h/2} {\left( {\sigma - \sigma_{0} } \right)_{\text{ex}} \left( {A_{\text{low}} (z) - A_{\text{high}} (z)} \right)} {\text{d}}z/hk_{\text{B}} T - \int\limits_{ - h/2}^{0} {\left( {\sigma - \sigma_{0} } \right)_{\text{in}} \left( {A_{\text{low}} (z) - A_{\text{high}} (z)} \right)} {\rm  d}z/hk_{\text{B}} T} \right) \hfill \\ \end{gathered} $$

Considering now only the leading order terms in $A_{t,r} (z)$ in either leaflet. In this case: one shall note $[A_{\text{low}} (z) - A_{\text{high}} (z)]_{\text{ex,in}} \sim [\delta A_{0} ]_{\text{ex,in}}$. Inserting the latter relation into Eq. 2, it follows at the leading order:

3 $$ L/L_{0} \sim \exp \left( { - \left( {\sigma - \sigma_{0} } \right)_{\text{ex}} [\delta A_{0} ]_{\text{ex}} /2k_{\text{B}} T - \left( {\sigma - \sigma_{0} } \right)_{\text{in}} [\delta A_{0} ]_{\text{in}} /2k_{\text{B}} T} \right) $$

Posing $\Updelta (\delta A_{0} ) = [\delta A_{0} ]_{\text{ex}} - [\delta A_{0} ]_{\text{in}}$, $\Upsigma (\delta A_{0} ) = [\delta A_{0} ]_{\text{ex}} + [\delta A_{0} ]_{\text{in}}$, $\left( {\sigma - \sigma_{0} } \right) = \left( {\sigma - \sigma_{0} } \right)_{\text{ex}} + \left( {\sigma - \sigma_{0} } \right)_{\text{in}}$ the surface tension of the membrane and $\left( {\Updelta \sigma - \Updelta \sigma_{0} } \right) = \left( {\sigma - \sigma_{0} } \right)_{\text{in}} - \left( {\sigma - \sigma_{0} } \right)_{\text{ex}}$ its difference in surface tensions Eq. 3 can be rewritten as:

4 $$ L/L_{0} \sim \exp \left( { - \frac{{\Upsigma \left( {\delta A_{0} } \right)}}{{2k_{\rm B} T}}\left( {\sigma - \sigma_{0} } \right) - \frac{{\Updelta \left( {\delta A_{0} } \right)}}{{2k_{\rm B} T}}\left( {\Updelta \sigma - \Updelta \sigma_{0} } \right)} \right) $$

Equation 4 is a generic equation relating allosteric changes to membrane tension. It is central to note that the model (Eq. 4) suggests NHE-1 as fully compliant to membrane biophysical properties. This is because no physical parameters associated with NHE-1 (such as compressibility) that have yet to be measured experimentally are introduced in Eq. 4.

The validity and coherence of Eq. 4 can now be evaluated against experimental data.

### Evaluation of Osmotic Shocks on NHE-1 Activation

Using optical techniques, it has been demonstrated that cells have a large reservoir of membrane [37] and an average membrane tension which can be remarkably low ($\sigma_{0} \sim 0.003\,{\text{mN}}/{\text{m}}$) [38], similar to the mean surface tension measured from in vitro systems (i.e., large liposomes where thermal undulations are dominant and dictate the mean membrane tension [39]). On the other hand, the difference in surface tensions between leaflets is much higher $\Updelta \sigma_{0} \sim - 0.9\,{\text{mN}}/{\text{m}}$ [21].

Let us assume that the mean surface tension is negligible. As the difference in the surface tensions between leaflets can then be related to the fluid phase vesicle radius: $R = - 8k_{\text{c}} /h\Updelta \sigma$ where $k_{\text{c}}$ is the membrane bending modulus (see Figs. 2b, c and appendix 2) [21], Eq. 4 can be rewritten as:

5 $$ L/L_{0} \sim \exp \left( {\frac{{\Updelta (\delta A_{0} )}}{{k_{B} T}}\frac{{2k_{c} }}{{hR_{0} }}\left[ {\frac{{R_{0} }}{R} - 1} \right]} \right) $$

It is noteworthy that as $L/L_{0} < 1$ one needs $\Updelta (\delta A_{0} ) < 0$. From Eq. 5, two results have to be notified. The first result is that it is possible to determine that moderate changes in the difference in surface tensions (and thus in the vesicle radius) will have an effect on NHE-1 allosteric activity only if $\frac{{\left| {\Updelta (\delta A_{0} )} \right|}}{{k_{\text{B}} T}}\frac{{2k_{\text{c}} }}{{hR_{0} }}\sim 1$. Assuming a vesicle radius $R_{0} \sim 50\,{\text{nm}}$, membrane thickness $h\sim 5\,{\text{nm}}$ and bending modulus $k_{\text{c}} \sim 2 \times 10^{ - 19} {\text{J}}$ [38], it follows: $\left| {\Updelta (\delta A_{0} )} \right|\sim 1\,{\text{nm}}^{2}$ at 37°C. The latter result suggests that if, between high and low affinity states, the differential surface of the protein and surrounding membrane required is around $\sim 1\,{\text{nm}}^{2}$, then slight changes in the difference in surface tensions can affect the allosteric state of the membrane protein. The second result is related to the membrane thickness. Because the membrane thickness appears in Eq. 5, this suggests that the allosteric switch may be influenced by agents that thicken or thin the membrane. However, because the bending modulus varies as $k_{\text{c}} \sim h^{2}$ (i.e., it is harder to bend a thick membrane than a thin one) Eq. 6 varies as: $L/L_{0} \sim \exp \left( { - h} \right)$ (the minus sign comes from the fact that $\Updelta (\delta A_{0} ) < 0$). Thus for similar lipid asymmetry, the allosteric switch would be less in thick membranes than in thinner ones.

As discussed in the Introduction, under moderate hypo-osmotic shocks (below an external dilution factor of ~30% with water) it was demonstrated in living cells that the size of membrane buds is affected. As vesicles budding from the membrane will take with them a different lipid asymmetry (see Fig. 2), the membrane lipid asymmetry (and related difference in surface tensions) will also change until it reaches a new equilibrium, which reflects the new size of the vesicle radius imposed by the osmotic pressure. Given a characteristic time for vesiculation $\sim 10\,{\text{ms}}$ [40, 41] the new equilibrium would appear within few seconds [42]. This model suggests that the osmotic pressure applied will have an effect on the membrane difference in surface tensions. The formula linking the osmotic pressure to the vesicle radius has been previously determined by others and is given by [43]:

6 $$ \Updelta \bar{P} = 2\frac{{1 - \bar{R}}}{{\bar{R}^{3} }} $$

**Fig. 3:**
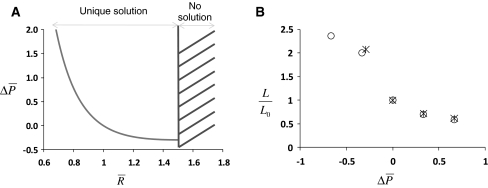
Comparison between theoretical and experimental $L_{0}$ values. **a** Graph representing the theoretical relationship between the radius of vesicles and the osmotic pressure [21, 33]. Note that no formal solution exists for extremely hypotonic values (*hashed region*). **b** Comparison of the values of the allosteric constant determined experimentally (*circle*) or calculated theoretically (*star*) for different osmotic pressures. Data are plotted as $L/L_{0}$ where $L_{0}$ is the allosteric constant value in isotonic conditions. Note that theoretical and experimental data are highly concordant. Theoretical values are not provided for hypotonic conditions where the theory does not provide formal solutions (equivalent to the *hashed region* in (**a**))

In Eq. 6 (plotted in Fig. 3a) $\Updelta \bar{P}$ represents the pressure difference between the inside and outside of cells normalised by the pressure inside cells in resting conditions and; $\bar{R}$ the ratio between the vesicle radius under osmotic pressure differences and the one in resting conditions (no pressure difference).

Therefore with Eqs. 4 and 5 it should be possible to determine how the NHE-1 allosteric switch is affected by hypotonic shocks. Data regarding the allosteric activation of NHE-1 under different conditions of tonicity were obtained [12] (Fig. 3b). Using Eqs. 5 and 6, we found that $\left| {\Updelta \left( {\delta A_{0} } \right)} \right|\sim 2.27 \pm 0.13\,{\text{nm}}^{2}$ was the optimal value to fit experimental data at 37°C (Table 1; Fig. 3b). The latter value is similar to our prediction done above on (i.e., $\left| {\Updelta \left( {\delta A_{0} } \right)} \right|\sim 1\,{\text{nm}}^{2}$) and corresponds to a characteristic length of $\sim 0.85\,{\text{nm}}$ that is in the range of Van der Waals radius for hydrogen or is similar to the size of ~3 hydrogen atoms covalently bound to NHE-1. Finally, assuming as done above that NHE-1 as a union of two cylinder-like monomers, this means that the allosteric switch occurs when the differential cross-sectional area of each NHE-1 monomer varies by ~10%.

**Table 1:** Comparisons between experimental data [12] and theory

Pressure	$\Updelta \bar{P}$	$L$	$L/L_{0}$ (Exp)	$L/L_{0}$ (Theory)	∆(%)
100	−0.66	3714	2.36	N/A	N/A
200	−0.33	3242	2	N/A	N/A
213	−0.29	N/A	N/A	2.08	N/A
300	0	1571	1	1	0
400	0.33	1107	0.70	0.71	1
500	0.66	929	0.59	0.61	2

Pressure values (*column one*) are given in mOsm. The last column “∆(%)” represents the relative errors expressed in percent. “N/A” indicates that this is an undefined variable either because Eq. 13 does not formally apply or, conversely that experimental data does not exist to describe NHE-1 allosteric activity

Note that we were not able to match the data using Eq. 4 with the mean surface tension considering the cell as an osmometer as the theoretical trend was opposed to experimental results (i.e., $L/L_{0} > 1$ with $\Updelta \bar{P} > 0$ and $L/L_{0} < 1$ with $\Updelta \bar{P} < 0$). Finally, this result strongly suggests that upon incubation of cells in hypertonic medium, it is the difference in surface tensions that is involved in NHE-1 allosteric activation and not the mean surface tension. This result solves the apparent paradox that, in intact cells, allosteric constants is going in opposite directions when cholesterol or hypertonic medium are used [13].

### Evaluation of the Effect of Symmetrical Changes in Mean Surface Tension on NHE-1 Allosteric Activation: Case of Cholesterol

Only the impact of the difference in surface tensions on NHE-1 allosteric activity has been discussed so far. Let us now determine the effect of a global but symmetrical change in the surface tension of each leaflet (i.e., at constant difference in surface tensions). This will enable us to compare the NHE-1 allosteric switch in either case.

Lipid asymmetry and changes in the mean surface tension have been demonstrated to have both an impact on the kinetics of membrane endocytosis [21, 44–46]. Accordingly, it should be possible to relate the changes in the kinetics of membrane endocytosis to those of surface tension. Intuitively, if the membrane is “pulled” laterally this means that the resulting tension will oppose any inward membrane budding; the converse is true when the membrane is “pushed” laterally, in which case the resulting tension will favour inward budding. Note that membrane buckling is not an option as the lipid asymmetry breaks the symmetry regarding the inward and outward membrane budding, to favour inward budding only.

In these conditions, namely at constant lipid asymmetry but in the presence of a tension, $\sigma = \sigma_{\text{in}} + \sigma_{\text{ex}}$, it is possible to demonstrate that the vesicle radius, *R*, is written as (see appendix 2):

7 $$ R \cong R_{0} \left( {1 + \frac{{h^{2} \sigma }}{{8k_{\text{c}} }}} \right) $$

In Eq. 7, $R_{0}$ corresponds to the unperturbed vesicle radius in the absence of surface tension. Recalling Eq. 4 and assuming initially that $\left( {\sigma_{0} } \right)_{\text{in}} = \left( {\sigma_{0} } \right)_{\text{ex}} = 0$, and that the lipid number asymmetry remains unchanged, Eq. 4 transforms to:

8 $$ L/L_{0} \sim \exp \left( {\frac{{\Upsigma (\delta A_{0} )}}{{k_{\rm B} T}}\frac{{4k_{\rm c} }}{{h^{2} }}\left[ {\frac{R}{{R_{0} }} - 1} \right]} \right) $$

**Fig. 4:**
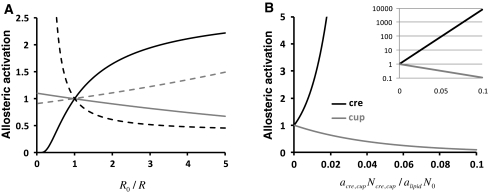
Predicted effects of surface tensions (**a**) and amphiphilic molecules (**b**) insertion in the membrane on NHE-1 allosteric constant ($L/L_{0}$). **a** Representation of Eq. 8 (*grey* effect of mean surface tension) and Eq. 5 (*black* effect of the difference in surface tensions) assuming $\Upsigma (\delta A_{0} )/k_{\text{B}} T \times 4k_{\text{c}} /h^{2} = - 0.1$ and $\Updelta (\delta A_{0} )/k_{\text{B}} T \times 4k_{\text{c}} /h^{2} = - 1$. *Dashed lines* represent the cases where $\Updelta (\delta A_{0} )$ and $\Upsigma (\delta A_{0} )$ are positives. **b** Representation of Eq. 11 at 37°C using the experimental determinations: $\Updelta \left( {\delta A_{0} } \right)\sim - 2\,{\text{nm}}$. The *inset* is scaled to represent cup-former effect

It is noteworthy that as $L/L_{0} < 1$ one needs $\Upsigma (\delta A_{0} ) < 0$. From Eq. 8 (plotted and compared to Eq. 5 in Fig. 4a), one can see that the relation to fluid phase endocytosis is totally different to that seen in Eq. 5 and it is possible to determine as above that the allosteric switch will be significantly affected only if $\frac{{\left| {\Upsigma (\delta A_{0} )} \right|}}{{k_{\text{B}} T}}\frac{{4k_{\text{c}} }}{{h^{2} }}\sim 1$, i.e. $\left| {\Upsigma (\delta A_{0} )} \right|\sim 0.1\,{\text{nm}}^{2}$ at 37°C. The last result suggests that if the opening of the membrane protein required between the relaxed and tensed states is 0.1 nm^2^, then slight changes in surface tension can favour a switch between states. The reason why $\Upsigma (\delta A_{0} )/\Updelta (\delta A_{0} )\sim 0.1$ lies in the fact that the ratio of factors within the exponential are also of the order 10: $\Upsigma (\delta A_{0} )/\Updelta (\delta A_{0} ) = h/R_{0} \sim 0.1$. Note also that in Eq. 8 appears the square of membrane thickness but as the bending modulus varies as $k_{\text{c}} \sim h^{2}$ the membrane thickness does not intervene in Eq. 9.

Cholesterol is a known modulator of NHE-1 allosteric activation. In particular it was demonstrated that cholesterol removal using methyl-β-cyclodextrin lowers the NHE-1 allosteric constant [13]. More specifically, it was shown that removing ~50% of membrane cholesterol leads to $L/L_{0} \sim 0.18$. Given that cholesterol molecules account for ~20–25% of lipid molecules [47] and partition equally between membrane leaflets, it should be possible to determine the changes in NHE-1 surface area (i.e., $\Upsigma \left( {\delta A_{0} } \right)$) leading to the allosteric switch. Let us further assume that the differences in surface tension of either leaflet are identical upon cholesterol removal (due to equal partitioning of cholesterol between leaflets). In these conditions the fraction of surface area removed is: $\Updelta N_{\text{chol}} \cdot a_{\text{chol}} /N_{0} \cdot a_{\text{lipid}}$, where *a* stands for the cross-section area of cholesterol ($a_{\text{chol}}$) or lipid ($a_{\text{lipid}}$), $N_{0}$ is the average number of lipids in membrane leaflets and $\Updelta N_{\text{chol}}$ the total amount of cholesterol in either leaflet. It follows a change in the membrane surface tension that is given by: $\sigma - \sigma_{0} \sim 2 \cdot \Updelta N_{\text{chol}} \cdot a_{\text{chol}} /N_{0} \cdot a_{\text{lipid}}$. In the last relation, the factor 2 ensures that both inner and out leaflet cholesterol are taken into consideration. As a result, Eq. 4 transforms to:

9 $$ L/L_{0} \sim \exp \left( { - 2K\Updelta N_{\text{chol}} /N_{0} \cdot a_{\text{chol}} /a_{\text{lipid}} \cdot \Upsigma (\delta A_{0} )/2k_{\text{B}} T} \right) $$

Providing an estimation for $a_{\text{chol}} /a_{\text{lipid}} \sim 22\,{\text{\AA}}/50\,{\text{\AA}}\, \sim 0.44$ [48], $K = 0.2\,{\text{mN}}/{\text{m}}$ [21] and $2 \times \Updelta N_{\text{chol}} /N_{0} = 0.5 \cdot 20\%$, one finds $\left| {\Upsigma \left( {\delta A_{0} } \right)} \right|\sim 5 \times 10^{3} \,{\text{nm}}^{2}$ at 37°C. This experimental result demonstrates a difference of four orders of magnitude with the deduction from the theory (Eq. 8). In this context the last figure is not realistic. As a result NHE-1 is very likely less compliant to symmetrical surface tension changes compared to difference in surface tensions (where a similar order of magnitude was deduced between the theory and experiments). Indeed, if the experimental deduction about $\Upsigma \left( {\delta A_{0} } \right)$ is higher than predicted this means that the physical effect of surface tension on NHE-1 is less than predicted (as these two parameters balance one another to predict the allosteric ratio). However, our results did not mention cholesterol rich micro-domains.

### Evaluation of the Effect of Cholesterol Rich Micro-Domains in NHE-1 Allosteric Activation

As a general rule, when cholesterol-rich membrane micro-domains are considered, the changes in NHE-1 allosteric activity is thought to result from membrane surface tension changes between tight/cholesterol-rich and fluid lipid phases [16, 49]. However, as seen above, the effect of surface tension on NHE-1 allosteric changes does not seem to be as important as the difference in surface tensions. As a result, when NHE-1 diffuses away from the micro-domain to the fluid lipid phase, the change in NHE-1 activity maybe the result of the existence of a difference in surface tensions in the fluid lipid phase that is not present in cholesterol-rich micro-domains.

The surface tension in cholesterol rich micro-domains is estimated at $\sigma_{0} \sim 0.5\,{\text{mN}}\,{\text{m}}^{ - 1}$ [38, 50]. Thus, the first term within the exponential function in Eq. 4 simplifies to: $- \Upsigma \left( {\delta A_{0} } \right)\left( {\sigma - \sigma_{0} } \right)/2k_{\text{B}} T\sim \Upsigma \left( {\delta A_{0} } \right)\sigma_{0} /2k_{\text{B}} T$ (where σ, the surface tension of the fluid lipid phase, is neglected).

Assuming that the difference in surface tensions within rafts is negligible, it follows that the second term within the exponential function in Eq. 4 simplifies to: $- \Updelta \left( {\delta A_{0} } \right)\left( {\Updelta \sigma - \Updelta \sigma_{0} } \right)/2k_{\text{B}} T\sim - \Updelta \left( {\delta A_{0} } \right)\Updelta \sigma /2k_{\text{B}} T$. With $\Updelta \sigma \sim 0.9{\text{mN}}\,{\text{m}}^{ - 1}$ [21]. As NHE1 is almost not affected by symmetrical surface tension changes, it follows that $\Upsigma \left( {\delta A_{0} } \right)\sigma_{0} < < \Updelta \left( {\delta A_{0} } \right)\Updelta \sigma$. As a result, the allosteric switch operating when NHE1 leaves rafts should be written as: $L/L_{0} \sim \exp \left( {\frac{{\Updelta (\delta A_{0} )}}{{k_{\text{B}} T}}\frac{{2k_{\text{c}} }}{{hR_{0} }}} \right)$. Our results suggest therefore the possibility of an allosteric switch when NHE-1 leave rafts not necessarily related to the membrane thickness but to the difference in surface tensions. Indeed, it is the differential opening of NHE-1 across the membrane that will prevail. In these conditions, using the above formula it is possible to deduce numerical values concerning the allosteric switch of NHE-1 when it diffuses away from rafts into the fluid lipid phase $\left( {L/L_{0} } \right)_{\text{fluid}} \sim 0.3$. A similar value of $\left( {L/L_{0} } \right)_{\text{fluid}} \sim 0.25$ was determined experimentally using methyl beta cyclodextrin in cells [13]. Thus, the presence or not of the difference in surface tensions may well be an important factor in the NHE-1 allosteric switch.

### Theoretical Comparisons Between Cup Formers and Crenators on NHE-1 Allosteric Switch

Amphiphile chemicals are classified into two groups, crenator or cup-former, based on their ability to change the cell membrane morphology [17]. Accurate theoretical and computer-based modelling have demonstrated that changes in the cell membrane morphology are related to the way amphiphiles affect the difference in surface tensions [51–53]. Crenators accumulate chiefly into the outer leaflet whereas cup-formers accumulate into the inner leaflet. As NHE-1 is also mechanically responsive to membrane accumulation of crenator and cup-former it is important to develop this last point. Noting $a_{\text{cup,cre}}$ and $N_{\text{cup,cre}}$ the cross-section area and membrane number of cup-formers and crenators and; $a_{\text{lipid}}$ and $\Updelta N_{0}$ the average cross-section area of lipid and the lipid number asymmetry in the membrane. It follows that the changes in NHE-1 activity are related to the difference in surface tensions when cup-formers or crenators are used are:

10a $$ \left\{ \begin{gathered} \left( \sigma \right)_{\text{ex}} = \left( {\sigma_{0} } \right)_{\text{ex}} \hfill \\ \left( \sigma \right)_{\text{in}} = - K\frac{{a_{\text{cup}} N_{\text{cup}} }}{{a_{\text{lipid}} N_{0} }} + \left( {\sigma_{0} } \right)_{\text{in}} \hfill \\ \end{gathered} \right. $$

10b $$ \left\{ \begin{gathered} \left( \sigma \right)_{\text{ex}} = - K\frac{{a_{\text{cre}} N_{\text{cre}} }}{{a_{\text{lipid}} N_{0} }} + \left( {\sigma_{0} } \right)_{\text{ex}} \hfill \\ \left( \sigma \right)_{\text{in}} = \left( {\sigma_{0} } \right)_{\text{in}} \hfill \\ \end{gathered} \right. $$

Inserting Eqs. 10a and 10b into Eq. 4 it follows:

11 $$ \left\{ \begin{gathered} \left( {\frac{L}{{L_{0} }}} \right)_{\text{cup}} \sim \exp \left[ {\frac{{\Updelta \left( {\delta A_{0} } \right) + \Upsigma \left( {\delta A_{0} } \right)}}{{2k_{\text{B}} T}}\frac{{a_{\text{cup}} N_{\text{cup}} }}{{a_{\text{lipid}} \Updelta N_{0} }}} \right] \hfill \\ \left( {\frac{L}{{L_{0} }}} \right)_{\text{cre}} \sim \exp \left[ { - \frac{{\Updelta \left( {\delta A_{0} } \right) + \Upsigma \left( {\delta A_{0} } \right)}}{{2k_{\text{B}} T}}\frac{{a_{\text{cre}} N_{\text{cre}} }}{{a_{\text{lipid}} \Updelta N_{0} }}} \right] \hfill \\ \end{gathered} \right. $$

When plotted in Fig. 4b this relation gives responses of NHE-1 to crenators and cup formers that follow the experimental results obtained when either Arachidonate or Chlorpromazine were tested on the allosteric regulation of NHE-1 [12].

## Discussion

NHE-1, mainly known as a pH regulator, has been shown to play an important role in biological mechanisms that involve modifications of cell shape and membrane composition. Indeed, this transporter is involved in cell volume regulation/motility/mitotic rounding [54–56] and has also been shown to play and important role in ischemia reperfusion [57, 58] and tumour progression [59, 60], two pathological situations in which cell lipids are modified. Conversely, fluid phase endocytosis that is paramount to control and maintain intracellular tonicity [61] is related to the lipid number asymmetry between leaflets that drives the difference in surface tensions needed for membrane budding. Previous works have estimated that the lipid asymmetry brings in a difference in surface area between membrane leaflets of ~4% [21]. Interestingly, this value is in the same order of magnitude to the one we found here to affect NHE-1 allosteric balance (~10%), as seen when changes in osmotic pressures are applied. This shows that NHE-1 is compliant to small differences in surface tensions. By contrast, we also found that the transporter is less compliant to the mean surface tension. This might rely on the fact that changes in the difference in surface tensions mean opposed, but symmetrical, effects in either leaflet. Therefore, the energy gained or lost in one leaflet would be balanced by a similar energy lost or gained in the other leaflet and the overall volume occupied by NHE-1 in the bilayer would not be strongly modified as well. In other words, changes in membrane asymmetry would impact NHE-1 allosteric transition for a low energy cost, whilst symmetric compression or stretching would be less efficient and more costly energetically.

The equations developed in this study can be used to yield an estimation of this NHE-1 symmetrical compression modulus.

From the equations developed in this study, it is possible to estimate this lateral symmetrical compression modulus of NHE-1 and compare to that of soluble proteins. We know that the compression modulus is expressed as$\chi \sim \Updelta V/V\Updelta P$, where *V* is NHE-1 volume and, $\Updelta P$ the pressure to apply to change NHE-1 volume by $\Updelta V$. The surface tension is dimensionally linked to the pressure via the membrane thickness $\Updelta \sigma \sim h\Updelta P$. Note that in this case, “∆σ” represents the symmetrical changes in the mean surface tension (and not the difference in surface tensions) of membrane leaflets. As from the cholesterol effect, we deduced that changing the mean surface tension by a factor $\Updelta \sigma = 2K\Updelta N_{\text{chol}} /N_{0} \cdot a_{\text{chol}} /a_{\text{lipid}} \sim 10^{ - 5} N\,m^{ - 1}$ allows a change in NHE-1 surface area by about $\Upsigma (\delta A_{0} )\sim 10^{3} \,{\text{nm}}^{2}$. As $A_{\text{NHE1}} \sim 11\,{\text{nm}}^{2}$ the values obtained are in the range of: $\chi_{\text{NHE1}} = h\Upsigma (\delta A_{0} )/A_{\text{NHE1}} \Updelta \sigma \sim 10^{2} \,{\text{Bar}}^{ - 1}$. This estimation is much higher than the order of magnitude of compressibility values found for aqueous proteins $\sim 10^{ - 6} - 10^{ - 5} \,{\text{Bar}}^{ - 1}$ [62–64]. To summarize, although lipid asymmetry is the dominant effect, NHE-1 is much more compressible to symmetrical pressure than soluble hydrated proteins.

Taken together, in building this model, we chiefly focused on the determination of surface tension energies involved without regarding boundary conditions at the protein level (e.g., lipid–protein mismatch and tension line). Of course, this model does not rule out the possible involvement of look-a-like “mismatch” between NHE-1 activity and membrane thickness. However, the results found in this study strongly suggest that the lipid asymmetry is likely to be paramount in the present mechanism.

It is important to note that the model exposed here that make full use of Eq. 6 can only explain small changes in osmotic pressures [33]. Many interesting studies have been published to understand how cells deal under large or extreme osmotic pressure differences [29, 65–67]. However, their conclusions are far beyond the scope of our study focused on small perturbations only.

An intriguing point, however, is the finding by Fuster et al. [16] that, using whole-cell patches, cholesterol activates NHE-1 whereas lyso-PC does the opposite. These results are symmetrical to our previous measurements in intact cells and to the theory developed in this study. One possible explanation that arises from this study is that the seal application of the patch pipette in the cell-attached configuration will produce important constraints and result in an inverted curvature that is equivalent to an inverted lipid asymmetry. Changes upon lipid or cholesterol addition would then be expected to produce opposite effects as those observed on intact cell membranes.

To summarize, this study shows that NHE-1, a mechanosensitive secondary transporter, is highly sensitive to membrane asymmetry, in contrast with widely studied mechanosensitive ion channels such as MSCL. Indeed, MSCL works as high-conductance emergency pressure valve that undergoes a very large conformational change upon opening [8–10]. In order to maintain the intracellular contents integrity in physiological conditions, this requires a very large energy barrier between the open and close conformations and very fast gating kinetics. By contrast, as NHE-1 exhibits transport rates that are orders of magnitude lower, its ability to modulate cell volume implies that it has to be much more compliant to membrane modification. This particular feature of NHE-1, which also emerged from the model presented in this study, is physiologically highly relevant.

## Appendix 1: Transition Between Protein States Mediated by Surface Tension

The model used (Eq. 1) is inspired from previous works by Cantor [22]. Let us consider a membrane protein in two possible states, $S_{1}$ and $S_{2}$. The cross-sectional areas of the protein in the transmembrane domain for each of the two states $A_{{S_{1} }} (z)$ and $A_{{S_{2} }} (z)$ vary with depth (denoted by the *z* axis) within the membrane. As a result, changes in protein conformation from $S_{1}$ to $S_{2}$ is accompanied with a difference in the protein cross-section area: $A_{{S_{2} }} (z) - A_{{S_{1} }} (z)$. The physical parameter conjugated to the surface area and that describes the forces that are applied from the membrane onto the protein along the *z* axis is the lateral pressure density: $p(z)$. If one defines a resting state of lateral pressure, $p_{0} (z)$, the pressure difference that leads to the transition between states is thus $\Updelta p(z) = p(z) - p_{0} (z)$.

Let us consider a set of membrane proteins that can be in two different states, $S_{1}$ and $S_{2}$. The chemical potentials of the protein in states $S_{1}$ and $S_{2}$ are written as:

12 $$ \begin{gathered} \mu_{{S_{1} }}^{{}} /RT = \mu_{{S_{1} }}^{0} /RT + \ln [S_{1} ] + (k_{\text{B}} T)^{ - 1} \int {p(z)A_{{S_{1} }}^{{}} } {\text{d}}z \hfill \\ \mu_{{S_{2} }}^{{}} /RT = \mu_{{S_{2} }}^{0} /RT + \ln [S_{2} ] + (k_{\text{B}} T)^{ - 1} \int {p(z)A_{{S_{2} }}^{{}} } {\text{d}}z \hfill \\ \end{gathered} $$

where *R* is the ideal gas constant and $[S_{i} ]$ the surface concentration of state $S_{i}$.

Noting $\Updelta \mu = \mu_{{S_{2} }}^{{}} - \mu_{{S_{1} }}^{{}}$ it follows that the lines of Eq. 12 can be rewritten as:

13 $$ \Updelta \mu /RT = \Updelta \mu_{{}}^{0} /RT + \ln [S_{2} ]/[S_{1} ] + (k_{\text{B}} T)^{ - 1} \int {p(z)(A_{{S_{2} }}^{{}} - A_{{S_{1} }}^{{}} )} {\text{d}}z $$

As one considers thermodynamic equilibrium, i.e. $\Updelta \mu = 0$, and that Eq. 13 is also true in resting conditions, i.e. when $p(z) = p_{0} (z)$, the unknown variable, $\Updelta \mu_{{}}^{0}$, can be determined and one finds finally:

14 $$ 0 = \ln [S_{2} ]/[S_{1} ] - \ln [S_{2} ]_{0} /[S_{1} ]_{0} + (k_{\text{B}} T)^{ - 1} \int {\left( {p(z) - p_{0} (z)} \right)\left( {A_{{S_{2} }} - A_{{S_{1} }} } \right)} {\text{d}}z $$

Equation 14 is similar to Eq. 1 and the starting point of our work.

## Appendix 2: Mean Surface Tension and Vesicle Radius

Fluid-phase endocytosis has been suggested to arise from the aminophospholipid translocase (flippase) triggering a phospholipid number asymmetry between the leaflets of cellular membrane [20, 21, 42, 68]. As already demonstrated elsewhere [21], the energy of a membrane patch budding of radius $R$, thickness $h$, and of neutral surface area $S_{0}$ can be described by the sum of three terms. The first term describing the motor force of budding associated with the endogenous difference of surface tension between the two leaflets of the plasma membrane and related to the phospholipid number asymmetry:$\Updelta \Upphi_{\Updelta \sigma } = \frac{{h\Updelta \sigma_{0} }}{2R}S_{0}$; where $\Updelta \sigma_{0} = - 2K\delta N_{0} /N_{0}$, *K* is the elastic modulus of leaflets (presumed to be identical for both in the first approximation), $\delta N_{0}$ the endogenous number of phospholipids in excess in the inner leaflet and $N_{0}$ the average phospholipid number in each leaflet. The second term of energy to take into consideration is the bending energy, which corresponds to the resistance to any curvature occurring during the membrane budding: $\Updelta \Upphi_{c} = 2k_{c} \frac{{S_{0} }}{{R^{2} }}$; where $k_{\text{c}}$ is the membrane bending modulus. Note that optimisation of both energies with regard to *R* provide: $R = - 8k_{\text{c}} /h\Updelta \sigma_{0}$. Finally, a third energy term can be added when the mean surface tension, $\sigma$, is considered: $\Updelta \Upphi_{\sigma } = \sigma S_{0} [1 + (R/4h)^{2} ]$. Optimising the new sum of these three energies with regard to *R* gives the solution we looked for namely Eq. 7. Note that in either cases the neutral surface area $S_{0}$ is supposed to flow and is not a limiting parameter of endocytosis [46].
